# Changes in dermoscopic photoaging and serum MMP-1 following 12 weeks of 5% lady finger banana peel extract nanoparticle serum: a single-arm proof-of-concept study

**DOI:** 10.3389/fmed.2026.1886006

**Published:** 2026-08-12

**Authors:** Khairina Nasution, Nelva Karmila Jusuf, Imam Budi Putra, Juliandi Harahap, Khairuddin Djawad, Ibnu Alferraly, Mustafa Mahmud Amin, Khairani Sukatendel

**Affiliations:** 1 Department of Dermatology and Venereology, Faculty of Medicine, Universitas Sumatera Utara, Medan, Indonesia; 2 Philosophy Doctor in Medicine Programme, Faculty of Medicine, Universitas Sumatera Utara, Medan, Indonesia; 3 Department of Community Medicine/Public Health, Faculty of Medicine, Universitas Sumatera Utara, Medan, Indonesia; 4 Department of Dermatology and Venereology, Faculty of Medicine, Universitas Hasanuddin, Makassar, South Sulawesi, Indonesia; 5 Department of Anatomic Pathology, Faculty of Medicine, Universitas Sumatera Utara, Medan, Indonesia; 6 Department of Psychiatry, Faculty of Medicine, Universitas Sumatera Utara, Medan, Indonesia; 7 Department of Obstetrics and Gynaecology, Faculty of Medicine, Universitas Sumatera Utara, Medan, Indonesia

**Keywords:** dermoscopy, facial photoaging, lady finger banana peel, matrix metalloproteinase-1, *Musa acuminata* colla, nanoparticle serum

## Abstract

**Background:**

Lady finger banana peel (*Musa acuminata* Colla) contains several phytochemical compounds with antioxidant activity that may be relevant to skin aging. However, clinical evidence regarding its use as a nanoparticle-based topical formulation remains limited.

**Objective:**

To evaluate within-participant changes in facial photoaging manifestations and serum matrix metalloproteinase-1 (MMP-1) levels following 12 weeks of topical application of a 5% lady finger banana peel extract nanoparticle serum.

**Methods:**

This prospective, single-center, single-arm, uncontrolled pretest–posttest proof-of-concept study enrolled women aged 25–50 years with clinical signs of facial skin aging. Participants applied 10 drops, approximately 0.5 mL, of the study serum once nightly for 12 weeks. Serum MMP-1 levels were measured using enzyme-linked immunosorbent assay at baseline and week 12. Dermoscopy Photoaging Scale (DPAS) scores were evaluated at baseline and weeks 4, 8, and 12. Changes in serum MMP-1 levels and DPAS scores were analyzed using the Wilcoxon signed-rank test and Friedman test, respectively. Adherence and adverse events were evaluated throughout the study.

**Results:**

Fifty participants were enrolled, of whom one discontinued the study because of non-adherence. A total of 49 participants completed the study and were included in the analysis. Mean serum MMP-1 levels decreased from 0.48 ± 0.39 ng/mL at baseline to 0.26 ± 0.25 ng/mL at week 12 (*p* < 0.001). Mean DPAS scores decreased from 12.69 ± 0.92 at baseline to 9.51 ± 0.77 at week 12 (*p* < 0.001). Changes in DPAS scores were modestly correlated with changes in serum MMP-1 levels (*r* = 0.347; *p* = 0.015). No adverse events were observed or reported among participants who completed the study.

**Conclusion:**

Lower DPAS scores and serum MMP-1 levels were observed following 12 weeks of topical serum use. Because this was an uncontrolled single-arm study, the findings should be regarded as preliminary and do not establish treatment efficacy, causality, systemic absorption, or modulation of cutaneous MMP-1. Randomized vehicle-controlled studies incorporating local skin biomarkers are required.

## Introduction

Skin aging is an inevitable, progressive, and multifactorial biological process that affects the appearance, structure, and function of the skin (1–5). It results from interactions between intrinsic and extrinsic factors. Intrinsic aging is influenced by genetic, ethnic, anatomical, and hormonal factors and generally begins in the mid-twenties, whereas extrinsic aging, or photoaging, is mainly associated with ultraviolet (UV) exposure, environmental pollution, smoking, poor nutrition, and other environmental factors. Clinically, photoaging is characterized by wrinkles, skin thinning, vascular changes, and irregular pigmentation (1, 5–8). Ultraviolet radiation induces reactive oxygen species, activates activator protein-1, and increases matrix metalloproteinase expression, particularly matrix metalloproteinase-1 (MMP-1). MMP-1 degrades type I and III collagen within the dermal extracellular matrix, thereby contributing to collagen fragmentation and wrinkle formation. MMP-1 has been widely implicated in collagen degradation and photoaging. However, circulating MMP-1 may not directly reflect its expression or enzymatic activity within facial dermal tissue (9–11).

Objective assessment is important for evaluating the severity of photoaging and monitoring changes during treatment. The Dermoscopy Photoaging Scale (DPAS), developed by Isik et al., is a non-invasive dermoscopic scoring system that quantitatively evaluates 11 facial photoaging features, including yellowish discoloration, yellow papules, white linear areas, lentigines, hypo-hyperpigmented macules, telangiectasia, actinic keratosis, senile comedones, superficial wrinkles, deep wrinkles, and criss-cross wrinkles (12, 13). Topical anti-aging formulations are widely used because of their convenience and direct application to affected skin areas. Plant-derived antioxidants have attracted particular interest because of their potential to reduce oxidative stress and attenuate signs of skin aging. Banana peel has traditionally been used as a natural skin-care ingredient and contains several phytochemical compounds with antioxidant activity (3, 14–17).

Lady finger banana peel (*Musa acuminata* Colla) contains polyphenolic compounds, including flavonoids and tannins, as well as alkaloids, saponins, terpenoids, anthocyanins, delphinidin, cyanidin, catecholamines, and carotenoids. These compounds may scavenge reactive oxygen species, reduce oxidative damage, suppress pathways associated with MMP-1 expression, and limit collagen degradation; however, these mechanisms are supported mainly by experimental and preclinical evidence and have not been directly established in human facial skin following topical application (16, 18–22). Nanoparticle-based delivery systems may improve the physicochemical stability, cutaneous distribution, and residence time of plant-derived bioactive compounds. Nevertheless, enhanced cutaneous penetration does not necessarily indicate systemic absorption, and pharmacokinetic evaluation, plasma measurement of active compounds, or direct skin-tissue assessment would be required to confirm systemic exposure or local modification of dermal MMP-1.

Circulating MMP-1 is a systemic biomarker and may not specifically reflect MMP-1 expression or enzymatic activity within facial skin because serum levels may also be influenced by systemic inflammation, metabolic activity, tissue remodeling, and other extracutaneous conditions. Therefore, serum MMP-1 was regarded in this study as an exploratory systemic biomarker rather than a direct measure of local cutaneous mechanisms. The present study aimed to evaluate within-participant changes in serum MMP-1 levels and DPAS scores following 12 weeks of topical application of a 5% lady finger banana peel extract nanoparticle serum. Because the study did not include a placebo, vehicle-control, untreated-control, or active-comparator group and did not incorporate randomization or blinding, it was conducted as a preliminary single-arm proof-of-concept study rather than a confirmatory efficacy trial.

## Methods

### Study design and ethical considerations

This was a prospective, single-center, single-arm, uncontrolled pretest–posttest proof-of-concept study conducted at the Dermatology and Venereology Outpatient Clinic of Prof. Chairuddin P. Lubis Universitas Sumatera Utara Hospital, Medan, Indonesia, from March 2025 to April 2026. All participants received the same intervention. No placebo, vehicle-control, untreated-control, or active-comparator group was included, and randomization and blinding were not performed.

The study was approved by the Research Ethics Committee of Universitas Sumatera Utara under approval number 269/KEPK/USU/2025 and by Prof. Chairuddin P. Lubis Universitas Sumatera Utara Hospital under approval number 2517/UN5.5.6. D2/PT.01.04/2025. Written informed consent was obtained from all participants before enrollment.

### Participants

Women aged 25–50 years with clinical signs of facial skin aging were recruited from the Dermatology and Venereology Outpatient Clinic. Facial skin aging was identified through medical history, physical examination, and dermatological examination.

Participants were excluded if they had facial dermatoses; a history of hypersensitivity to facial cosmetic products; use of topical anti-aging products containing retinoids, antioxidants, vitamin C, vitamin E, or alpha-hydroxy acids within the previous month; use of oral isotretinoin, antioxidants, vitamin C, or vitamin E; facial rejuvenation procedures within the previous 3 months; hormonal contraceptive or hormone-replacement therapy; relevant systemic disease or malignancy; pregnancy; breastfeeding; or menopause.

### Sample-size calculation

The minimum sample size was calculated using the formula for a paired numerical comparison described by Dahlan (23). The expected standard deviation of 3.9 and minimum clinically relevant mean difference of 2.63 were derived from Muslim et al. (15). Using a two-sided *α* of 0.05 and *β* of 0.05, corresponding to 95% power, the calculation yielded a minimum of 29 participants. After allowing for an anticipated dropout rate of 20%, the minimum required sample size was 35 participants. Fifty participants were enrolled to ensure an adequate final sample.

### Participant withdrawal criteria

1. Did not apply the serum for 3 consecutive days.
2. Applied the serum for less than 11 weeks in total.
3. Used oral or topical anti-aging products or underwent facial rejuvenation procedures during the study period.

### Preparation of the plant material and extract

Fresh lady finger banana peels (*Musa acuminata* Colla) were collected from Deli Serdang Regency, North Sumatra, Indonesia. Botanical identification was performed at the Laboratory of Plant Systematics, Herbarium Medanense, Universitas Sumatera Utara, under identification number 2619/MEDA/2024.

The peels were separated from the fruit pulp, washed under running water, and dried at room temperature or in an oven at 40–50 °C until dry and brittle. The dried material was pulverized and passed through a 60-mesh sieve to obtain uniform simplicia powder. The powder was macerated with 96% ethanol for three consecutive 24-h cycles with intermittent stirring. The combined filtrates were concentrated using a rotary evaporator at approximately 40 °C. The concentrated extract was stored in a tightly closed container at 4 °C until further use.

### Antioxidant activity assay

Antioxidant activity was evaluated using the 2,2-diphenyl-1-picrylhydrazyl radical-scavenging assay. A 0.5 mM DPPH solution was prepared by dissolving 10 mg of DPPH in methanol to a final volume of 50 mL. The maximum absorption wavelength was determined at 515.5 nm.

The extract was tested at concentrations of 30, 60, 90, and 120 ppm. Each extract solution was mixed with the DPPH solution and incubated for 30 min at room temperature in the dark. Absorbance was subsequently measured using a UV–visible spectrophotometer.

Radical-scavenging activity was calculated using the following equation:


$$\begin{array}{l} \text{Radical}-\text{scavenging activity}\;(\%)\\ =[(\text{Acontrol}-\text{Asample})/\text{Acontrol}]\times 100 \end{array}$$

The inhibitory concentration required to reduce 50% of DPPH radical activity was calculated from the linear regression equation relating extract concentration to percentage inhibition.

### Preparation of the nanoparticles

Lady finger banana peel extract nanoparticles were prepared using the ionic gelation method. A 0.2% w/v chitosan stock solution was prepared in 1% w/v acetic acid, whereas a 0.1% w/v sodium tripolyphosphate solution was prepared in distilled water. The extract stock solution was prepared by dissolving 300 mg of concentrated extract in 1 mL of 70% ethanol.

Six milliliters of chitosan solution were mixed with 1 mL of sodium tripolyphosphate solution and 1 mL of extract stock solution. The mixture was stirred using a magnetic stirrer at 1,500 rpm for 10 min to induce ionic gelation. The suspension was subsequently sonicated for 10–15 min to reduce particle size and improve system homogeneity.

### Preparation of the serum formulation

The topical serum was prepared using a carbopol-based vehicle. The final formulation contained 5% v/v lady finger banana peel extract nanoparticle suspension, 0.5% carbopol, 0.2% triethanolamine, 0.05% butylated hydroxytoluene, 7.5% propylene glycol, 0.5% methylparaben, 0.01% propylparaben, and distilled water to a final volume of 100 mL.

Carbopol was dispersed in distilled water and allowed to hydrate for approximately 24 h. Methylparaben and propylparaben were dissolved in propylene glycol and subsequently incorporated into the carbopol dispersion. Butylated hydroxytoluene was added as an antioxidant. The nanoparticle suspension was gradually incorporated under gentle stirring until a homogeneous formulation was obtained. Triethanolamine was added incrementally to obtain a pH of approximately 5–6 and the desired serum consistency.

### Rationale for the 5% concentration

The 5% w/v concentration was selected as an exploratory formulation concentration based on preliminary formulation feasibility, acceptable physicochemical characteristics, and the antioxidant activity of the extract. A formal dose-ranging or concentration-response study was not performed; therefore, the selected concentration should not be interpreted as an established optimal anti-aging dose.

### Particle-size characterization

Particle-size distribution was evaluated using a laser-diffraction Particle Size Analyzer (ANALYSETTE 22 NanoTec/MicroTec Plus, FRITSCH, Germany) based on Mie scattering theory. The measurement was performed over a particle-size range of 0.01–42.30 μm. The differential volume distribution, dQ3(x), and cumulative volume distribution, Q3(x), were recorded. The mean, median, and modal particle diameters were obtained from the instrument-generated analysis.

### Physical evaluation and preliminary stability

The serum formulation was evaluated for organoleptic characteristics, homogeneity, pH, spreadability, viscosity, and adhesion. Preliminary physical stability was evaluated using a cycling test consisting of storage at 4 °C and 40 °C for 24 h at each temperature for six cycles. Changes in color, odor, homogeneity, pH, viscosity, and spreadability were recorded after each cycle.

The cycling test was performed as a preliminary assessment of physical stability and was not intended to establish the formal shelf life of the formulation.

### Intervention

Participants were instructed to apply 10 drops, equivalent to approximately 0.5 mL, of the 5% lady finger banana peel extract nanoparticle serum evenly over the facial skin once daily at night for 12 weeks after cleansing with a hypoallergenic facial cleanser. The periocular and perioral areas were avoided.

All participants were provided with the same sunscreen formulation with a sun protection factor of 30 and were instructed to apply it regularly every morning, avoid excessive sun exposure, and refrain from using other topical or oral anti-aging treatments or undergoing facial rejuvenation procedures during the study period.

### Serum MMP-1 assessment

Venous blood samples were collected at baseline and week 12. Serum MMP-1 concentrations were measured using a Human MMP-1 ELISA Kit, catalog number EHMMP1, according to the manufacturer’s instructions. Serum MMP-1 concentrations were expressed in ng/mL.

### Dermoscopic assessment

Facial photoaging was evaluated using the Dermoscopy Photoaging Scale with an ILLUCO IDS-1100 dermoscope at baseline and weeks 4, 8, and 12. The forehead, right malar area, left malar area, and chin were examined.

The DPAS evaluates 11 dermoscopic features, including yellowish discoloration, yellow papules, white linear areas, lentigines, hypo-hyperpigmented macules, telangiectasia, actinic keratosis, senile comedones, superficial wrinkles, deep wrinkles, and criss-cross wrinkles. Each feature was scored as present or absent in each facial region, resulting in a total score ranging from 0 to 44. Higher scores indicated more severe photoaging.

### Adherence and participant withdrawal

Adherence was assessed through participant interviews, review of daily treatment records, and scheduled follow-up visits at weeks 4, 8, and 12. Participants were withdrawn if they did not apply the serum for three consecutive days, used the serum for less than 11 weeks, used another topical or oral anti-aging product, or underwent a facial rejuvenation procedure during the study period.

### Safety assessment

At each follow-up visit, participants were interviewed and underwent dermatological examination for potential adverse events, including skin dryness, desquamation, erythema, edema, stinging, burning sensation, and pruritus. Adverse events were documented and graded as absent, mild, moderate, or severe.

### Statistical analysis

Statistical analyses were performed using SPSS software. Continuous variables were summarized as mean ± standard deviation or median and interquartile range, as appropriate. Normality was evaluated using the Shapiro–Wilk test.

Serum MMP-1 levels at baseline and week 12 were compared using the Wilcoxon signed-rank test. Changes in DPAS scores across baseline and weeks 4, 8, and 12 were evaluated using the Friedman test, followed by Wilcoxon signed-rank tests for pairwise comparisons. The association between changes in serum MMP-1 levels and changes in DPAS scores was evaluated using Spearman correlation analysis, with a bootstrapped 95% confidence interval. A two-sided *p* < 0.05 was considered statistically significant. Pairwise comparisons were adjusted for multiple testing using the Bonferroni method, with an adjusted significance threshold of *p* < 0.0083.

### Clinical trial registration

The study was not prospectively registered in a public clinical trial registry. This has been acknowledged as a limitation of the study.

## Results

### Participant flow

**Figure 1:**
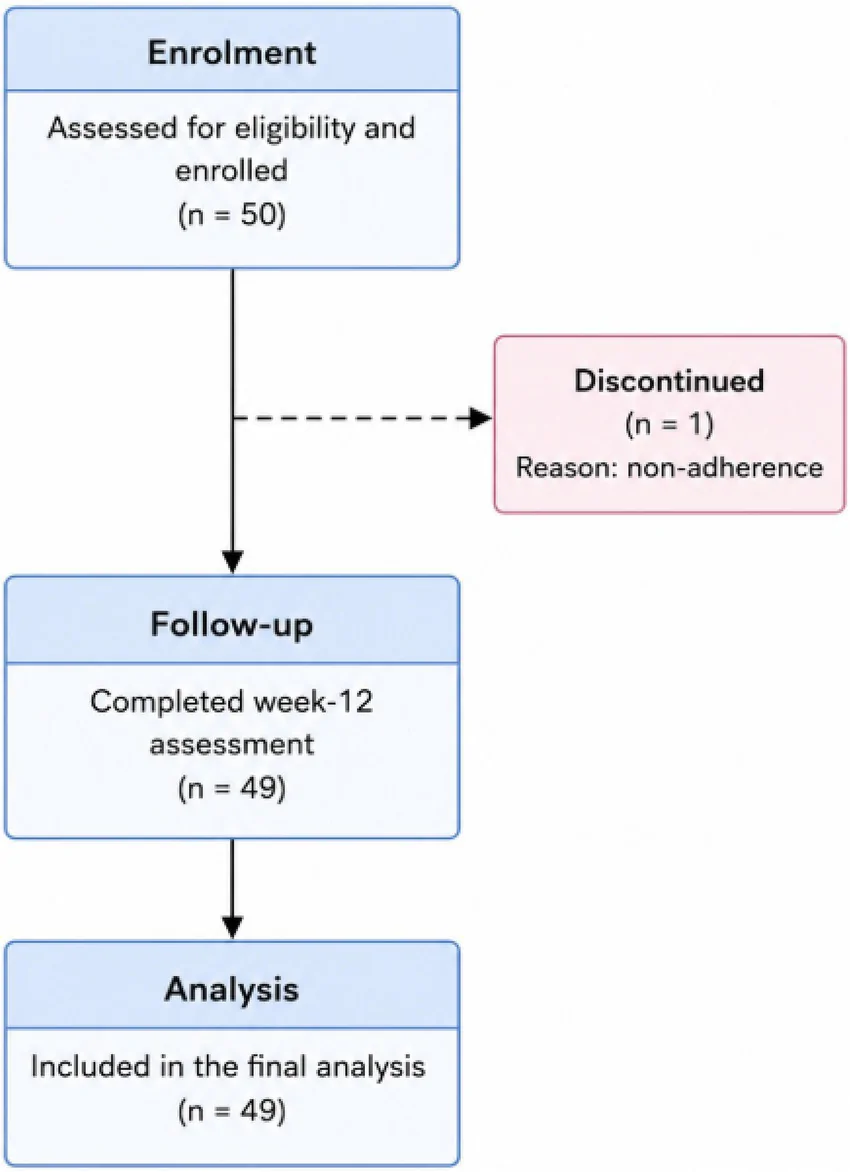
Flow of participants through the study.

A total of 50 participants were enrolled. One participant discontinued the study because she did not apply the study serum for more than three consecutive days. Therefore, 49 participants completed the 12-week intervention and were included in the final analysis. No additional losses to follow-up occurred (Figure 1).

### Participant characteristics

The mean age of the 49 participants included in the analysis was 36.96 ± 5.22 years. The largest age group was 35–39 years, comprising 20 participants (40.8%), followed by 40–44 years, comprising 14 participants (28.6%).

### Particle-size characteristics

**Figure 2:**
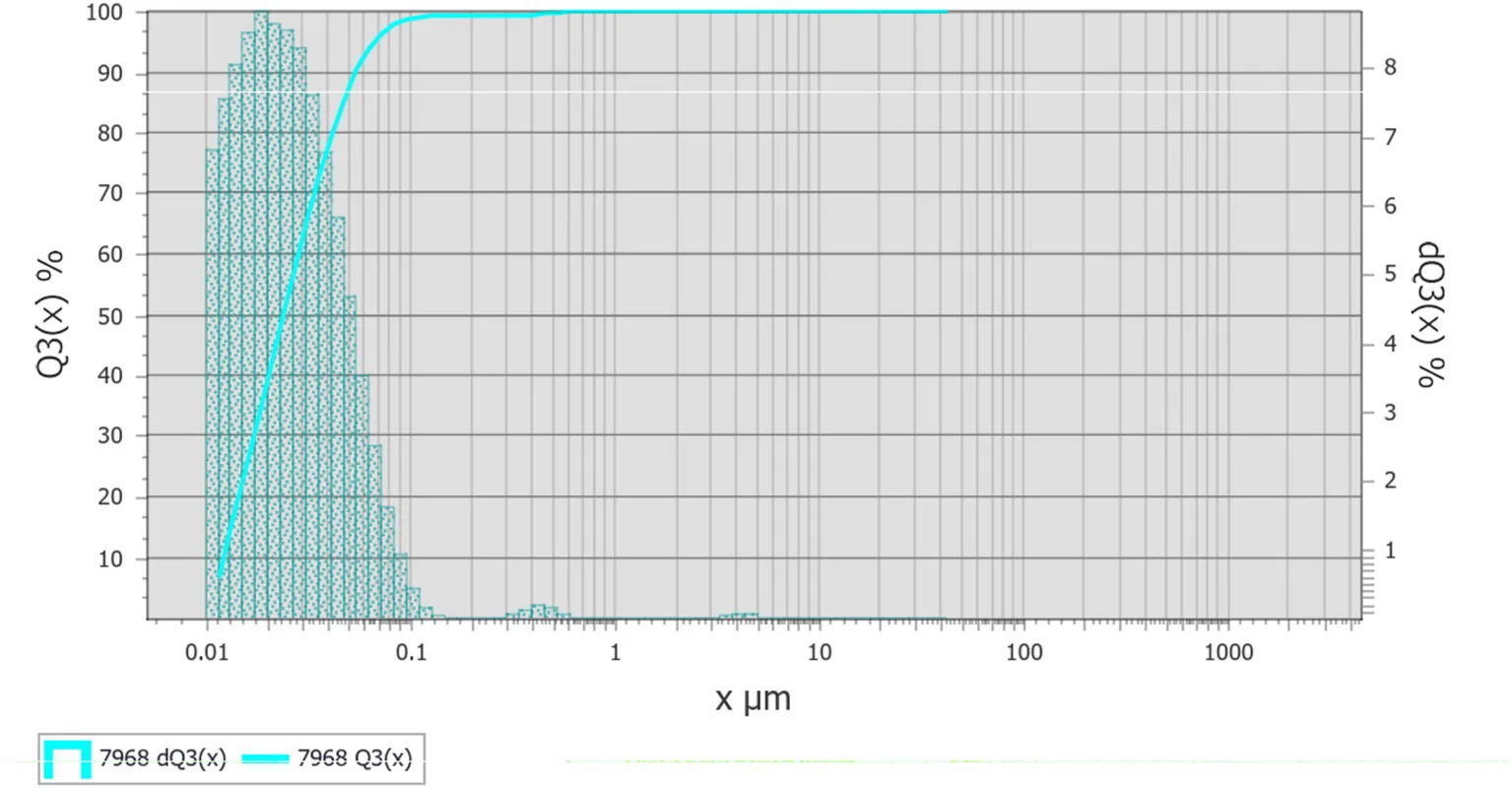
Particle-size distribution of the 5% lady finger banana peel extract nanoparticle serum measured using laser diffraction. The histogram represents the differential volume distribution, *dQ3(x)*, whereas the solid line represents the cumulative volume distribution, *Q3(x)*.

Laser-diffraction analysis indicated a particle-size distribution with a predominant population in the nanoscale range. The mean particle diameter was 37.58 nm, while the median and modal particle diameters were 24.00 and 11.36 nm, respectively. Minor particle populations were also observed at larger particle sizes (Figure 2).

### Antioxidant activity

Lady finger banana peel extract demonstrated DPPH radical-scavenging activities of 10.70, 24.87, 56.37, and 73.71% at concentrations of 30, 60, 90, and 120 ppm, respectively. The calculated IC₅₀ value was 71.84 ppm (Table 1).

**Table 1:** Distribution of participants based on age.

Age (years)	*n*	%
25–29	4	8.2
30–34	10	20.4
35–39	20	40.8
40–44	14	28.6
45–49	1	2
Total	49	100

### Serum MMP-1 levels

As shown in Table 2, mean serum MMP-1 levels were lower at week 12 than at baseline, decreasing from 0.48 ± 0.39 ng/mL to 0.26 ± 0.25 ng/mL (*p* < 0.001, Wilcoxon signed-rank test).

**Table 2:** Comparison of serum MMP-1 levels before and after the application of 5% lady finger banana peel (*Musa acuminata* Colla) extract nanoparticle serum.

Time point	*N*	MMP-1 (ng/mL)	*P*
		Mean ± SD	
Baseline	49	0.48 ± 0.39	<0.001*
Week 12	49	0.26 ± 0.25	

* Wilcoxon.

### DPAS scores

As shown in Table 3, mean DPAS scores decreased across the 12-week observation period, from 12.69 ± 0.92 at baseline to 11.71 ± 0.89 at week 4, 11.04 ± 0.89 at week 8, and 9.51 ± 0.77 at week 12 (*p* < 0.001, Friedman test).

**Table 3:** Comparison of DPAS scores before and after the application of 5% lady finger banana peel (*Musa acuminata* Colla) extract nanoparticle serum.

Time point	*n*	DPAS	*p*
		Mean ± SD	
Baseline	49	12.69 ± 0.92	<0.001*
Week 4	49	11.71 ± 0.89	
Week 8	49	11.04 ± 0.89	
Week 12	49	9.51 ± 0.77	

* Friedman.

Pairwise analyses demonstrated statistically significant differences between each assessment time point. Lower DPAS scores were observed beginning at week 4 and continued through week 12.

Further analysis was performed to evaluate differences in DPAS scores between measurement time points. As shown in Table 4, Pairwise comparisons demonstrated significantly lower DPAS scores at each subsequent assessment time point. All six unique comparisons remained statistically significant using the Bonferroni-adjusted significance threshold of *p* < 0.0083.

**Table 4:** Pairwise comparison of DPAS scores between observation periods.

Comparison	*p*-value
Baseline vs. week 4	<0.001*
Baseline vs. week 8	<0.001*
Baseline vs. week 12	<0.001*
Week 4 vs. week 8	<0.001*
Week 4 vs. week 12	<0.001*
Week 8 vs. week 12	<0.001*

* Wilcoxon signed-rank test. Using the Bonferroni correction for six pairwise comparisons, statistical significance was defined as *p* < 0.0083.

Spearman correlation analysis demonstrated a statistically significant but modest positive correlation between changes in DPAS scores and changes in serum MMP-1 levels (*r* = 0.347; *p* = 0.015; bootstrapped 95% CI, 0.070–0.590). Change scores for serum MMP-1 and DPAS were calculated as baseline values minus week-12 values, so that positive values represented reductions during the study period.

### Adherence and safety

One participant was withdrawn because of non-adherence. All 49 participants included in the final analysis completed the scheduled assessments.

No adverse events were observed or reported among the participants who completed the study. Specifically, no participant developed skin dryness, stinging, erythema, burning sensation, or pruritus during the 12-week study period. No serious adverse events occurred.

## Discussion

In this single-arm proof-of-concept study, serum MMP-1 levels and DPAS scores were lower following 12 weeks of topical use of a 5% lady finger banana peel extract nanoparticle serum. A statistically significant but modest positive correlation was also observed between changes in DPAS scores and changes in serum MMP-1 levels. These findings represent within-participant changes and should not be interpreted as confirmatory evidence of treatment efficacy or causality because the study did not include a placebo, vehicle-control, untreated-control, or active-comparator group.

Particle-size analysis demonstrated a mean diameter of 37.58 nm, with median and modal diameters of 24.00 and 11.36 nm, respectively. These findings support the presence of a predominantly nanoscale particle population in the formulation. Nanoparticle-based topical delivery systems may improve the physicochemical stability, cutaneous distribution, and residence time of plant-derived bioactive compounds. However, the presence of nanoscale particles does not itself establish enhanced skin penetration, systemic absorption, or superior clinical efficacy. These properties would require additional permeability, release, and pharmacokinetic studies.

The 5% concentration used in the present study referred to 5% w/v nanoparticle suspension incorporated into the final serum formulation, rather than 5% dry banana peel extract or 5% dried nanoparticles. Therefore, the concentration should be interpreted as a formulation concentration of the nanoparticle suspension. Because a formal dose-ranging or concentration-response study was not performed, this concentration should not be considered an established optimal anti-aging dose.

The ethanol extract of lady finger banana peel demonstrated antioxidant activity, with an IC₅₀ value of 71.84 ppm. Antioxidants play an important role in neutralizing reactive molecules and protecting cells from oxidative stress caused by intrinsic and environmental factors. Topically applied antioxidants have also been reported to improve skin appearance, reduce inflammatory responses, delay manifestations of skin aging, and provide protection against ultraviolet-induced skin damage (24, 25). Nevertheless, the antioxidant activity demonstrated in an *in vitro* DPPH assay does not directly establish antioxidant activity within human facial skin after topical application.

DPAS scores decreased progressively throughout the 12-week observation period, with changes already observed at week 4. This finding suggests that dermoscopic manifestations of photoaging changed during the study period. DPAS is a simple, non-invasive dermoscopic scoring system that evaluates photoaging features that may not be clearly visible to the naked eye. It includes 11 dermoscopic criteria (yellowish discoloration, yellow papules, white linear areas, lentigines, hypo-hyperpigmented macules, telangiectasia, actinic keratosis, senile comedones, superficial wrinkles, deep wrinkles, and criss-cross wrinkles) assessed across the forehead, right malar area, left malar area, and chin (13, 26, 27).

The observed reduction in DPAS scores is consistent with previous studies evaluating topical anti-aging interventions using dermoscopic assessment. Muslim et al. (15) reported a significant reduction in DPAS scores following application of a 3% purple passion fruit seed (*Passiflora edulis* Sims var. edulis) extract cream. Mazzeo et al. (28) also reported a reduction in median DPAS scores after 16 weeks of topical application of a formulation containing 0.8% piroxicam and sunscreen. In addition, Tran et al. (29) described a gradual reduction in total DPAS scores among participants receiving 0.1% adapalene cream. These findings support the usefulness of DPAS for monitoring changes in facial photoaging manifestations during topical treatment.

However, the reduction in DPAS scores in the present study cannot be attributed exclusively to the banana peel extract. All participants were instructed to use sunscreen regularly and avoid excessive sun exposure throughout the study. Photoprotection may independently limit ongoing ultraviolet-induced skin damage and influence several components of the DPAS. Repeated clinical monitoring, standardized facial cleansing, discontinuation of other anti-aging treatments, and changes in participants’ skin-care behavior may also have contributed to the observed clinical changes. Because no sunscreen-only or untreated-control group was included, the independent contributions of the serum and photoprotection could not be determined.

The serum vehicle may also have influenced the clinical findings. The formulation contained a carbopol-based vehicle, propylene glycol, and other excipients that may affect skin hydration, barrier function, spreadability, and overall cosmetic appearance. Therefore, without a vehicle-control group, the observed changes cannot be separated into effects attributable to the banana peel extract, the nanoparticle delivery system, the serum base, sunscreen use, or their combined effects.

Skin aging is characterized by progressive loss and fragmentation of collagen fibers within the dermis, contributing to wrinkle formation, skin laxity, and reduced viscoelasticity. MMP-1 is a collagenase involved in the degradation of type I and III collagen, which are major components of the dermal extracellular matrix. Increased MMP-1 expression may accelerate collagen fragmentation, disrupt extracellular matrix integrity, and impair the skin’s ability to maintain and repair its structure. Ultraviolet radiation and environmental pollution may further increase MMP-1 activity and accelerate extracellular matrix damage. Accordingly, strategies targeting oxidative stress, collagen synthesis, and matrix metalloproteinase activity have been investigated as potential approaches to preventing or reducing manifestations of skin aging (30–32).

The lower serum MMP-1 levels observed at week 12 may be biologically relevant; however, serum MMP-1 is a circulating biomarker and is not equivalent to MMP-1 expression or enzymatic activity within facial dermal tissue. Circulating MMP-1 may be influenced by systemic inflammation, tissue remodeling, metabolic activity, and other extracutaneous processes. Consequently, the observed serum MMP-1 change cannot be interpreted as direct evidence that the topical formulation inhibited MMP-1 activity within facial skin.

The modest correlation between changes in DPAS scores and serum MMP-1 levels indicates that participants with greater changes in one parameter tended to demonstrate changes in the other. Nevertheless, this association does not establish causality, mediation, or a direct biological pathway. Therefore, the findings should not be interpreted as evidence that the reduction in serum MMP-1 caused or mediated the improvement in dermoscopic photoaging features.

Previous experimental studies have demonstrated that plant-derived antioxidants may suppress MMP-1 expression under ultraviolet- or oxidative-stress conditions. Extracts derived from banana leaf (*Musa sapientum* L.), brocolli flower (*Brassica oleracea* L. var. italica Plenck), *Syzygium aromaticum* L., Seungma-Galgeun-Tang, and *Panax ginseng* Meyer have been reported to reduce MMP-1 expression in human dermal fibroblasts exposed to UVB or oxidative stress (33–37). Yoo et al. (37) specifically reported that a banana-derived extract reduced MMP-1 expression in human skin fibroblasts. These findings support a biologically plausible role for plant-derived phytochemicals in reducing oxidative stress and extracellular matrix degradation. However, the cited studies primarily involved cellular or experimental models and cannot be considered direct confirmation of the mechanism observed in the present clinical study.

Lady finger banana peel contains several phytochemical compounds, including polyphenols, flavonoids, tannins, and carotenoids, that may scavenge reactive oxygen species and reduce oxidative damage. Such local antioxidant activity could theoretically suppress signaling pathways involved in MMP-1 expression and collagen degradation (16, 18–22, 38). Nevertheless, this proposed mechanism was not directly investigated. The present study did not include skin biopsy, cutaneous MMP-1 measurement, local oxidative-stress markers, procollagen assessment, or dermal fibroblast analysis. Therefore, the local antioxidant and MMP-1–modulating mechanisms remain biologically plausible but unconfirmed.

The nanoparticle delivery system may have affected the cutaneous distribution and stability of the extract. Nanoparticle-based topical formulations have been reported to improve the stability of active compounds, prolong their residence time, and facilitate delivery into specific skin layers (39–42). However, enhanced cutaneous penetration does not necessarily indicate systemic absorption. The present study did not measure plasma concentrations of the extract’s active constituents, transdermal absorption, or pharmacokinetic parameters. It therefore remains unknown whether any bioactive compounds reached the systemic circulation at biologically relevant concentrations.

Particle-size distribution was characterized using laser diffraction, which demonstrated a mean particle diameter of 37.58 nm. However, the polydispersity index was not obtained because it was not reported by the analytical method used. Zeta potential, particle morphology, encapsulation efficiency, drug loading, release profile, and skin-permeation characteristics were also not evaluated. Therefore, although the particle-size findings supported the presence of a predominantly nanoscale particle population, the dispersion uniformity, surface properties, and delivery performance of the nanoparticle system could not be fully characterized.

No local adverse events were observed or reported among participants who completed the 12-week study, suggesting that the formulation was well tolerated during the observation period. However, the sample size and follow-up duration were insufficient to exclude uncommon, delayed, or long-term adverse effects. In addition, the formulation underwent only preliminary physical stability assessment using a six-cycle temperature cycling test. Formal accelerated and long-term stability studies were not performed; therefore, the shelf life and long-term physicochemical and microbiological stability of the serum cannot be established from the present data.

This study has several limitations. Its single-center, single-arm design did not include randomization, blinding, or a control group, increasing the potential for observer, expectation, temporal, and behavioral biases. Sunscreen use represented an important co-intervention, while the serum vehicle may also have independently influenced the clinical appearance of the skin. Serum MMP-1 was used as an exploratory systemic biomarker rather than a direct measure of cutaneous MMP-1, and no skin-tissue, permeability, or pharmacokinetic assessments were performed. The 5% w/v concentration referred to the nanoparticle suspension incorporated into the final serum formulation; because it was not compared with other concentrations, it should not be considered an established optimal dose. The study included only women and had a follow-up period of 12 weeks, limiting generalizability and evaluation of long-term clinical outcomes and safety. Although laser-diffraction analysis supported the presence of a predominantly nanoscale particle population, with a mean particle diameter of 37.58 nm, additional characterization, including polydispersity index, zeta potential, particle morphology, encapsulation efficiency, drug loading, release profile, and skin-permeation properties was not performed. In addition, the absence of formal accelerated and long-term stability studies precluded determination of the formulation’s shelf life and long-term physicochemical and microbiological stability.

Despite these limitations, the study provides preliminary clinical and systemic biomarker observations that may inform the design of future controlled investigations. Further studies should employ randomized, double-blind, vehicle-controlled designs; standardize sunscreen use across study groups; incorporate blinded outcome assessment; compare multiple formulation concentrations; conduct comprehensive nanoparticle, permeation, and stability characterization; and include local skin biomarkers such as cutaneous MMP-1, procollagen expression, or other indicators of extracellular matrix remodeling. The present findings therefore support further investigation of the formulation but do not establish its efficacy or biological mechanism as a topical anti-aging treatment.

## Conclusion

Following 12 weeks of topical use of a 5% lady finger banana peel extract nanoparticle serum, lower DPAS scores and serum MMP-1 levels were observed compared with baseline. A modest association was also identified between changes in these parameters. No adverse events were observed during the 12-week study period.

Because this was an uncontrolled single-arm proof-of-concept study, the findings do not establish treatment efficacy, causality, systemic absorption, or modulation of MMP-1 within facial skin. Randomized vehicle-controlled trials incorporating local skin biomarkers and comprehensive formulation characterization are required to confirm the clinical effects and biological mechanisms of the formulation.

## Data Availability

The original contributions presented in the study are included in the article/supplementary material, further inquiries can be directed to the corresponding author.
